# Case Report: Identification of Potential Prognosis-Related *TP53* Mutation and *BCL6-LPP* Fusion in Primary Pituitary Lymphoma by Next Generation Sequencing: Two Cases

**DOI:** 10.3389/fendo.2021.673908

**Published:** 2021-07-26

**Authors:** Yi Zhang, Liyuan Ma, Jie Liu, Huijuan Zhu, Lin Lu, Kan Deng, Wenbin Ma, Hui Pan, Renzhi Wang, Yong Yao

**Affiliations:** ^1^ Department of Neurosurgery, Peking Union Medical College Hospital, Beijing, China; ^2^ Department of Endocrinology, Peking Union Medical College Hospital, Beijing, China

**Keywords:** primary pituitary lymphoma, *TP53*, *BCL6*, high-dose methotrexate, diffuse large B cell lymphoma, next generating sequencing

## Abstract

**Background:**

Primary pituitary lymphoma (PPL) is an extremely rare disease with poor prognosis. Although PPL has been shown to be different from classical primary central nervous system lymphoma because of the embryological origin of structures, individual and precise treatment of PPL remains unknown.

**Methods:**

A 61-year-old man and a 65-year-old woman both diagnosed with primary pituitary diffuse large B cell lymphoma underwent genetic analysis of cerebrospinal fluid and tumor tissue by next generation sequencing.

**Results:**

In the first case, partial remission was achieved following R²-MTX chemotherapy. In the other case with *TP53* mutation and *BCL6*-*LPP* fusion, disease progressed although different chemotherapy regimens were given.

**Conclusion:**

The gene mutation of *TP53* and *BCL6* may be identified as a marker responsible for prognostic difference in patients with PPL. Genetic analysis may provide a novel approach for precise management and prognosis prediction.

## Introduction

Primary pituitary lymphoma (PPL) is an extremely rare clinical entity with much poorer prognosis, while it has an emerging trend these years (1, 2). It is commonly limited in the sellar and parasellar areas without systematic involvement. Histologically, B-cell lymphoma is the most common cell type, followed by T cell type and NK/T cell type (1). The diagnosis of PPL usually can only be determined by pathological analysis since the clinical history, radiological findings and physical examinations do not show significant distinctions with other intracranial neoplasms (1). Although previous studies tend to consider PPL as different primary central nervous system lymphoma (PCNSL) because of embryological origin, the treatment of PPL often follows the management protocol of PCNSL. However, we noticed that the sensitivity and effectiveness of treatment varied in the patients of PPL (3–5). Genetic analysis might provide a novel approach to selecting the most appropriate regimen and predicting the prognosis of PPL patients. Here we reported two cases of PPL undergoing genetic analysis of cerebrospinal fluid (CSF) and tumor tissue by next generation sequencing (NGS). We firstly found that the gene mutation of *TP53* and *BCL6* might be responsible for prognostic difference in patients with PPL.

## Material and Methods

CSF was collected in Streck tubes (Streck, Omaha, NE, USA) from each patient for lymphoma gene detection. Tumor tissue samples and peripheral blood as controls were collected for Whole Exome Sequencing (WES). Library construction was performed using protocols of the Illumina TruSeq DNA library preparation kit (Illumina, San Diego, CA), and then hybridized to custom-designed biotinylated oligonucleotide probes (Roche NimbleGen, Madison, WI, USA). Targeted sequencing was carried out using Illumina HiSeq 3000 platform (Geneplus, Beijing, China). Reads were further aligned to human genome (hg19) using Burrows–Wheeler Aligner (BWA, version 0.7.12-r1039). Somatic single nucleotide variants (SNVs), small insertions and deletions (InDels) and fussion were detected using GATK toolkit (version 3.4). CNV(copy number variation) were identified by CNVKit (version: 0.9.2.dev0).

### Case Presentation

#### Case 1

**Figure 1:**
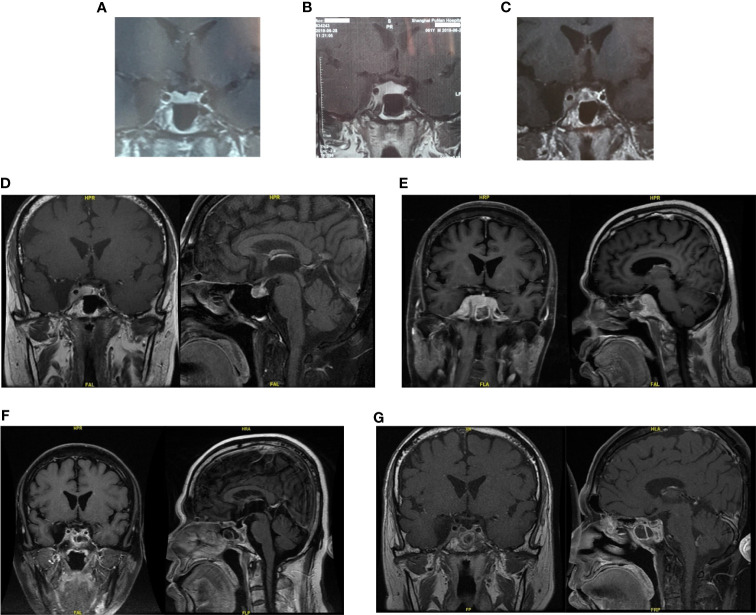
The pituitary images in different period of Case 1. **(A, B)** Pituitary magnetic resonance imaging (MRI) 10 and 5 months ago both demonstrated suspected “pituitary microadenoma” on the right side. **(C)** Three months ago, enhanced MRI showed the mass in sella turcica enlarged to 1.3 × 0.9 cm, involving the right cavernous sinus and internal carotid artery. **(D)** One months ago, the pituitary MRI showed the mass might be “pituitary macroadenoma” (12.7 × 6.7 × 11.1 mm, demonstrating equal T1 and equal T2 signal with slightly homogeneous enhancement) in the sellar region with the right cavernous sinus invasion (Knosp IV). **(E)** Pituitary MRI reexamination half months after biopsy surgery showed the mass enlarged compared with before, involving bilateral cavernous sinus. **(F, G)** Enhanced head MRI + DWI and Pituitary gland plain scan + enhanced MRI showed the mass was significantly smaller than before after three months’ chemotherapy.

A 61-year-old man suffered from increasing right-side headache and blurred vision for 10 months and right eye lid ptosis for 4 months. Laboratory tests, including blood routine and biochemistry, immunological indices, thorax, abdominal and pelvis CT and lumbar puncture were normal. Endocrine investigation disclosed multiple anterior pituitary hypofunction: adrenocorticotrophic hormone of 21.34 pg/ml, morning cortisol of 40.6 nmol/L (NR: 166–507 nmol/L); Thyroid function: FT3 3.09 ng/L, FT4 0.31 ng/dL, TSH 0.11 μIU/ml; Gonadal hormone: LH 1.0 mIU/ml, FSH 2.5 IU/ml, E <18 pmol/L, P <0.159 nmol/L, testosterone <0.087 nmol/L, DHEA 0.1 μmol/L, prolactin of 792 mIU/L (NR: 166–507 nmol/L); GH 1.2 ng/ml. Pituitary magnetic resonance imaging (MRI) 10 and 5 months ago both demonstrated suspected “pituitary microadenoma” on the right side (**Figures 1A, B**). The patient was twice diagnosed with “autoimmune hypophysitis” and treated with hydrocortisone or prednisone together with azathioprine, and twice diagnosed with “viral encephalitis” and received pulsed methylprednisolone in different local hospitals in these 10 months. However, his symptoms didn’t improve but worsened. The MRI three months ago showed the mass in sella turcica enlarged to 1.3 × 0.9 cm, involving the right cavernous sinus and internal carotid artery (**Figure 1C**). The remaining past medical, personal and family history was unremarkable.

Last month, the patient visited our hospital. Blood test work up showed hypothalamus–pituitary dysfunction similar with before. The pituitary MRI showed the mass might be “pituitary macroadenoma” (12.7 × 6.7 × 11.1 mm, demonstrating equal T1 and equal T2 signal with slightly homogeneous enhancement) in the sellar region with the right cavernous sinus invasion (Knosp IV) (**Figure 1D**). Physical examination after admission revealed right third cranial nerve palsy and classic signs of Cushing’s syndrome resulting from glucocorticoids treatment.

A presumptive diagnosis of nonsecretory pituitary macroadenoma was made, and the mass caused anterior pituitary hypofunction. Endoscopic endonasal transsphenoidal surgery was performed for biopsy in our hospital. Pathological examination confirmed diffuse large B cell lymphoma (DLBCL) (germinal center-like). Immunohistochemical staining showed the tumor cells to be immunoreactive for B-cell marker CD20 and negative for the T-cell marker CD3. Further marker studies showed the tumor cells to be positive for Bcl-2, Bcl-6, CD5, CD20, C-MYC (index 60%) and P53. Markers Mum-1, CD10, CD23, CD3, Cyclin D1, CD30 (Ki-1), AE1/AE3, CAM5.2, ER, PIT-1, T-PIT were negative. Cell proliferation index Ki-67 was 70%. *In situ* hybridization showed EBER ISH (−).

A total body PET/CT, bone marrow biopsy and CSF analysis confirmed the absence of systemic involvement. Testing for the HIV were negative.

Thus, the diagnose of primary central nervous system lymphoma (DLBCL, GCB type, double positive expression, Ann Arbor stage IE A) was made. Further genetic testing for lymphoma by NGS of CSF sample showed the patient contained *MYD88* (c.794T>C), *TNFRSF14* (c.95C>T), *ETV6* (c.26G>A) and *ETV6* (c.33+1G>A) mutations (**Table 1**). NGS result of tumor tissue was similar to it.

**Table 1:** Gene mutations of cerebrospinal fluid sample in two patients.

Gene	Transcript	Mutation	Amino acid	Function Zones	Variation frequency
***Case 1***					
*MYD88*	NM_002468.4	c.794T>C	p.L265P	EX5	5.0%
*TNFRSF14*	NM_003820.2	c.95C>T	p.A32V	EX2	4.7%
*ETV6*	NM_001987.4	c.26G>A	p.S9N	EX1	1.6%
*ETV6*	NM_001987.4	c.33+1G>A	–	IVS1	1.6%
***Case 2***					
*MYD88*	NM_002468.4	c.794T>C	p.L265P	EX5	55.8%
*TP53*	NM_000546.5	c.401T>G	p.F134C	EX5	39.0%
*CD79B*	NM_000626.2	c.68-1G>C	–	IVS1	23.7%
*PCLO*	NM_033026.5	c.6632C>T	p.T2211I	EX5	11.7%
*JAK2*	NM_004972.3	c.678G>T	p.R226S	EX7	3.6%
*JAK2*	NM_004972.3	Amplification	–	9p24.1	14.2
*CD274*	NM_014143.3	Amplification	–	9p24.1	13.6
*PDCD1LG2*	NM_025239.3	Amplification	–	9p24.1	7.1
*BCL6-LPP*	NM_001706.4 /NM_001167672.1	Fusion	–	3q27.3/3q28	38.4%

Pituitary MRI reexamination half months after biopsy surgery showed the mass enlarged compared with before, involving bilateral cavernous sinus (**Figure 1E**). Three courses of R²-MTX chemotherapy (800 mg iv d1 of Rituximab, 7 g iv d2 of methotrexate, and 25 mg d1–14 of lenalidomide) were administrated in the next three months. MRI scan showed marked reduction of the tumor size (**Figures 1F, G**). The symptom of eyelids ptosis, blurred vision and headache gradually improved. He is considered to be in partial remission.

#### Case 2

A 65-year-old woman had paroxysmal headache with no obvious inducement from 2 months ago, accompanied with nausea, denying vomiting, blurred vision or slurred speech. The patient also had dry mouth, polydipsia and polyuria with loss of appetite and fatigue. For past medical history, she received an operation of right adnexectomy and total hysterectomy because of mucinous cystadenoma of right ovary 10 years ago.

**Figure 2:**
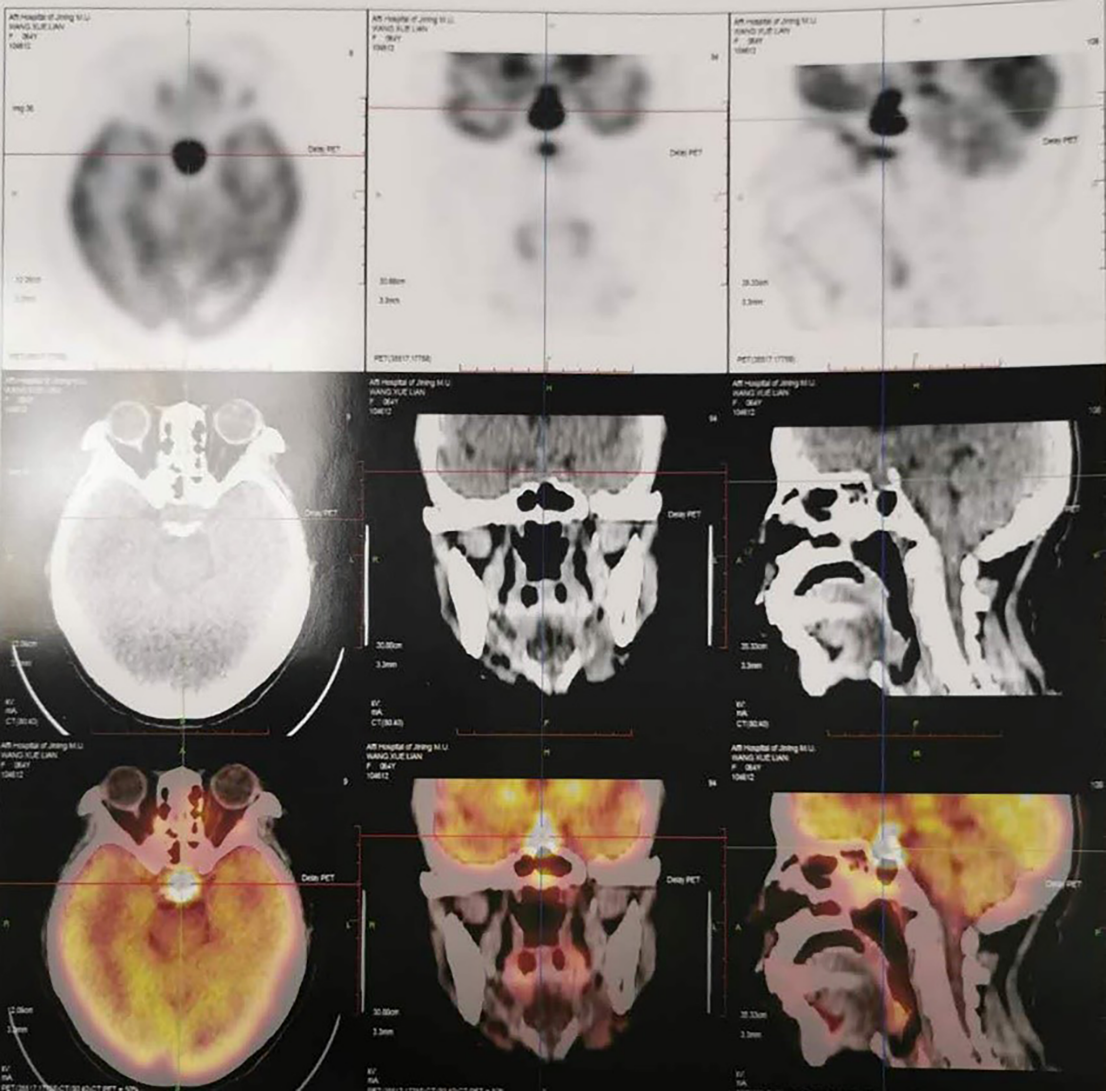
PET-CT one month ago showed the mass (2.3 × 2.0 cm) in pituitary gland with increased FDG uptake and SUVmax of 75.7 in Case 2.

Last month in local hospital, endocrines test showed hypopituitarism: FT4 8 pmol/L, TSH 0.11 mIU/ml; LH <0.01 mIU/ml, FSH 1.16 mIU/ml; 0 ACTH 1.88 pmol/L, serum cortisol 0.506, 8 ACTH 1.91 pmol/L, serum cortisol 0.536; GH 0.803 ng/ml, IGF-1 150 ng/ml. The tumor marker NSE was of 21.77 ng/ml (0–16.3), while AFP and CEA were normal. IgG4 was of 0.47 g/L. Pituitary MRI showed a mass appeared as soft tissue density in sellar area with a size of about 1.5 × 1.3 × 2.2 cm, and partial of it had no clear boundary between optic chiasma. PET-CT showed the mass (2.3 × 2.0 cm) in pituitary gland with increased FDG uptake and SUVmax of 75.7 (**Figure 2**). The patient was treated orally with desmopressin 0.05 mg bid, prednisone acetate 5 mg qd, and euthyrox 25 μg qd. The symptoms of thirst and polyuria were obviously relieved, but headache and nausea were not relieved.

Two weeks ago, the headache worsened with persistent pain, accompanied by nausea and vomiting. She went to the department of emergency in our hospital with a blood pressure of 85/66 mmHg. After symptomatic treatment, the patient was admitted to the department of neurosurgery. Physical examination revealed normal vision and visual field. Further examination was finished, which showed: TSH 30.014 μIU/ml, FT3 1.55 pg/ml; Tumor markers: CEA 2.3 ng/ml, CA 125 10.9 μ/ml; IgG4 506 mg/L, ESR 44 mm/h.

Mass in sellar area which might be metastatic pituitary cancer was considered and endoscopic endonasal transsphenoidal surgery was performed for biopsy. Pathological examination showed the tumor was consistent with DLBCL (B cell derived from germinal center). The immunohistochemical staining showed: CD20(++), Bcl-2(+), Mum-1(10%+), Bcl-6(95%+), C-MYC(80%+), P53(+), CD3(scattered+), CD5(scattered+), CD15(−), CD10(−), CD30(Ki-1)(−), PAX-5(+), AE1/AE3(−), CgA(−), NUT(−), HMB45(−), Syn(−), S-100(−), Ki-67(index 90%). *In situ* hybridization showed EBER ISH (−).

**Figure 3:**
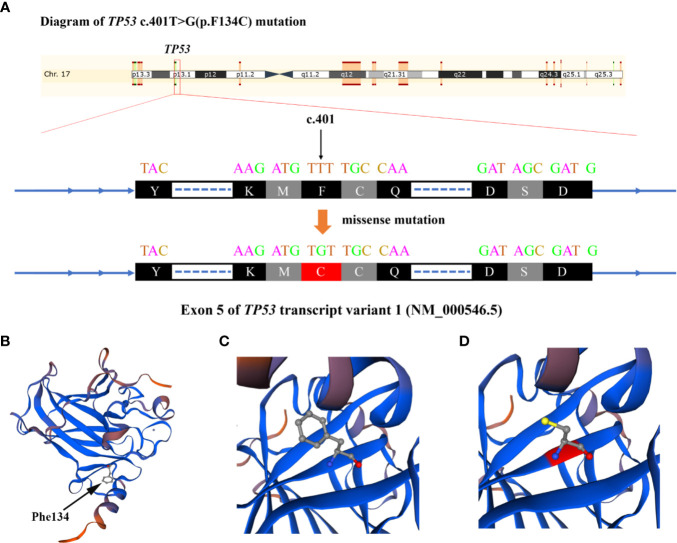
Diagram of *TP53* c.401T>G (p.F134C) alteration in Case 2. **(A)** The base of c.401 mutated from T to G and amino acid changed from phenylalanine to cysteine. **(B)** The 3-dimentional model structure of TP53 wild-type analyzed by SWISSMODEL. **(C)** The Phe134 of TP53. **(D)** The Cys134 of mutated TP53.

**Figure 4:**
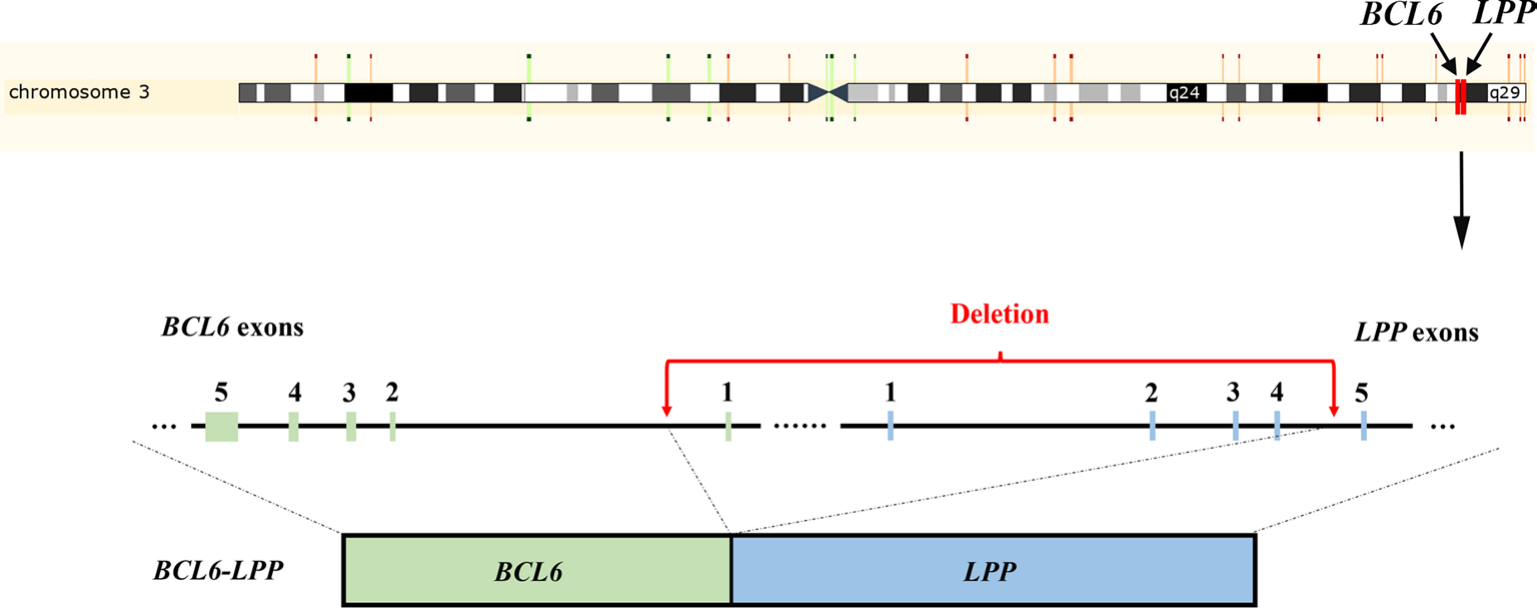
Schematic representation of the *BCL6-LPP* fusion in Case 2. An 838 kb-sized deletion of chromosome 3q27.3–3q28 (base 187461439 on chromosome 3q27 to base 188299507 on chromosome 3q28), resulting in a fusion of the *BCL6* with the *LPP* gene.

NGS testing for lymphoma-related genes of CSF sample showed the patient contained point mutations of *MYD88* (c.794T>C), *TP53* (c.401T>G), *CD79B* (c.68-1G>C), *PCLO* (c.6632C>T) and *JAK2* (c.678G>T), amplification of gene *JAK2*, *CD274*, *PDCD1LG2* and fusion of gene *BCL6-LPP* (**Table 1**). **Figures 3** and **4** schematically display two mutations closely related to poor prognosis of lymphoma, *TP53* (c.401T>G) mutation and *BCL6-LPP* fusion, which existed in patient 2 but not in patient 1. Result of WES of tumor tissue was consistent with CSF.

**Figure 5:**
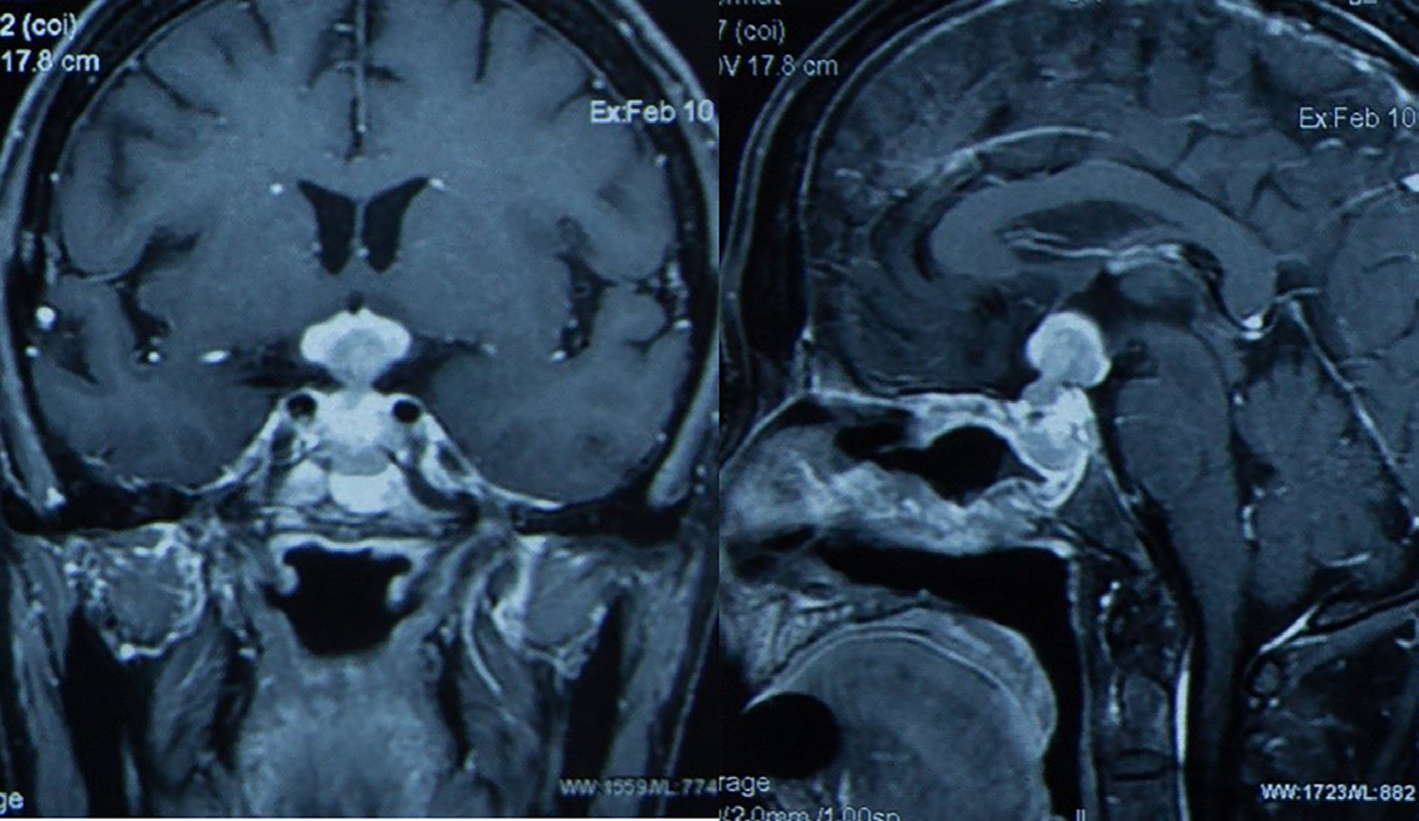
Pituitary MRI at two months from the beginning of chemotherapy demonstrated the tumor (size of about 2 × 1.5 × 3.2 cm) was larger than before without cavernous sinus involving in Case 2.

CT of the chest, abdomen, and pelvis as well as bone marrow biopsy were negative for dissemination. Then the patient received chemotherapy in local hospital, including one course of R-HDMTX (rituximab and high-dose methotrexate) chemotherapy regimen, one course of rituximab associated to temozolomide and one course of R-MT protocol (580 mg d0 of rituximab, 2.0 g d1 of methotrexate, and 200 mg d2-5 of temozolomide). A pituitary MRI at two months from the beginning of chemotherapy demonstrated the tumor (size of about 2 × 1.5 × 3.2 cm) was larger than before without cavernous sinus involving (**Figure 5**). Thus, the treatment of chemotherapy continued.

## Discussion

We present two cases of PPL using genetic analysis to guide treatment and predict prognosis. To the best of our knowledge, this is the first attempt to distinguish PPL from PCNSL genetically and manage PPL based on gene sequencing and we administered different treatment modality of chemotherapy and targeted therapy accordingly. We also identified that gene mutation of *TP53* and *BCL6* was responsible for prognostic difference. Normally, the treatment of PPL often follows the management protocol of PCNSL consisting of surgery, chemotherapy and/or radiotherapy (6). However, it is said that surgical intervention suggests no obvious benefits in the outcome of PCNSL and the neurotoxic effects of radiotherapy should be noted (3, 5). Therefore, the therapeutic regimen consisting of HD-MTX combined with rituximab and other cytostatic drugs that penetrate the blood–brain barrier is highly recommended (3, 4). Both of our patients initiated chemotherapy immediately after the diagnosis of PPL was confirmed pathologically, but one patient seemed not to be sensitive to such regimen. Genetic analysis was thus performed to predict prognosis and adjust treatment modality. Since the two patients showed different prognosis, we identified gene mutation of *TP53* and *BCL6* as a distinct characteristic after genetic comparison was made. Some other typical gene mutations of PPL are also investigated here which we believe are highly likely to make a difference.

*TP53* is mutated in 20–25% of aggressive B-cell lymphoma. The negative prognostic impact of *TP53* mutations in DLBCL has been reported in a number of studies (7). Mutation and copy loss of *TP53* are independent negative prognostic factors in DLBCL (8), while recent studies indicated that the prognostic role should be validated when combined with other indexes (9). For instance, Dobashi et al. found that *TP53* mutations and *TP53* deletions were confirmed to be poor prognostic factors for overall survival (OS) and progression-free survival (PFS) only when both aberrations co-existed (10). In the patients of DLBCL treated with R-CHOP, *TP53* mutation significantly correlate with worse survival in either ABC- or GCB-DLBCL (11). Such a negative correlation could also be seen in chemotherapy ± rituximab (CCT-treated) PCNSL patients, with hotspot/direct DNA contact *MUT*-*TP53* being predictive of poor outcome (12). Todorovic et al. investigated that *TP53* was the only gene harboring mutations in all surveyed PCNSL patients, which showed a more frequent mutation incidence than DLBCL (13). Therefore, we believe the unfavorable prognostic effect of *TP53* mutation is more likely to be employed in PCNSL and PPL patients. *TP53* alterations can either give rise to a loss-of-function or a gain-of-function phenotype (14). In the cases of *TP53* loss-of-function, they might undergo two sequential events. The first event is mutation or methylation of the *TP53* promoter, leading to appearance of a cell with increased risk of malignant transformation. The second event is the loss of an intact allele of the gene; this change is necessary for tumorigenesis (15). The gain-of-function mutations could partly account for the observation of *TP53* overexpression in haematological malignancies and resistance to conventional chemotherapeutic agents, leading to poor survival (14). The drug resistance could be seen in studies pointing out that response to both CHOP and R-CHOP treatments was significantly inferior in patients with *TP53* mutation and that the Hodgkin Reed-Sternberg cell lines with drug resistance all contained *TP53* defects (7, 14). One of our patients showed *TP53* mutation without sufficient drug effect and prognosis was not well, which might indicate a potentially predictive role of *TP53* in the prognosis of PPL.

In DLBCL, The *BCL6* locus can fuse with different partner genes. Ueda et al. found that non-immunoglobulin/*BCL6* gene fusion in DLBCL is a poor prognostic indicator and plays a pathogenetic role in a proportion of DLBCL (16). In PCNSLs, the *BCL6* gene fusion with its partner genes such as lipoma-preferred partner (*LPP*) in band 3q27, may contribute to aberrant expression of *BCL6* protein (17). A deletion in 3q leads to loss of an 837-kb fragment extending from the first intron of *BCL6* to the third intron of the *LPP* gene, which may bring the *BCL6* gene under the control of regulatory elements of the *LPP* gene or the miRNA-28 gene located in intron 4 of *LPP* (17). One of our patients showed *BCL6-LPP* gene fusion and the prognosis was not good. Whether BCL6 can be considered as a prognostic factor of PCNSL is still controversial. Cady et al. investigated that *BCL6* was associated with inferior OS alone or concomitant with del (6) (q22) (18). However, the expression of *BCL6* was paradoxically correlated with the prognosis of PCNSL. Kreher et al. found that *BCL6* expression was associated with shorter Progression-free survival (PFS) (19), while Lossos et al. found *BCL6* expression had improved survival (20) and Niparuck et al. found that *BCL6* expression showed no significant predictive effect in PFS and OS (21). Considering the high relative expression of *BCL6* can be detected in the majority of PCNSL cases, a potential role for *BCL6* antagonists in the next generation of therapies for PCNSL could be explored (22).

## Conclusion

Generally, we presented two extremely rare cases of PPL and developed genetic analysis as a novel approach for prognosis prediction and treatment adaption where gene mutation of *TP53* and *BCL6* were identified as a marker for prognostic difference of PPL. We believe such an attempt will inspire clinicians to yield more precise and effective management approaches of PPL and more details about genetic analysis in PPL should be validated in larger prospective studies.

## Data Availability Statement

The data presented in the study are deposited in the NCBI Sequence Read Archive (SRA) repository, accession numbers (SRR14774001, SRR14774002, SRR14783994, SRR14783995, SRR14783996).

## Ethics Statement

The ethical approvals for this study were granted by the PUMCH Institutional Review Board. The patients/participants provided their written informed consent to participate in this study.

## Author Contributions

Conception and design: YY, RW, and YZ. Development of methodology: HP, WM, and YZ. Acquisition and analysis of data: YZ, LM, JL, and KD. Writing, review and revision of manuscript: YZ, LM, and JL. Technical and material support: HJZ and LL. Study supervision: YY, RW, and HJZ. All authors contributed to the article and approved the submitted version.

## Funding

This study was supported by Chinese Academy of Medical Sciences Innovation Fund for Medical Sciences (No. 2016-I2M-1-002) from YY: providing conception and design and supervision. Youth Science Foundation of Peking Union Medical College Hospital (No. pumch201911867) from YZ: analysis of data and writing manuscript.

## Conflict of Interest

The authors declare that the research was conducted in the absence of any commercial or financial relationships that could be construed as a potential conflict of interest.

## Publisher’s Note

All claims expressed in this article are solely those of the authors and do not necessarily represent those of their affiliated organizations, or those of the publisher, the editors and the reviewers. Any product that may be evaluated in this article, or claim that may be made by its manufacturer, is not guaranteed or endorsed by the publisher.

## Glossary

**Table:**

ABC-DLBCL	activated B-cell-like lymphoma-DLBCL
ACTH	adrenocorticotropic hormone
AFP	alpha-fetoprotein
Bcl-2	B-cell lymphoma 2 protein
*BCL6*	B-cell lymphoma 6 gene
CA125	carbohydrate antigen 125
*CD79B*	Cluster of Differentiation 79B gene
CEA	carcinoembryonic antigen
CgA	chromogranin-A
CNV	copy number variation
CSF	cerebrospinal fluid
DHEA	Dehydroepiandrosterone
DLBCL	diffuse large B cell lymphoma
E	estrogen
EBER ISH	*in situ* hybridization of EBV-encoded RNA
ER	Estrogen receptor
ESR	erythrocyte sedimentation rate
*ETV6*	translocation-Ets-leukemia virus 6 gene
FDG	fluorodeoxyglucose
FSH	follicle-stimulating hormone
FT3	free triiodothyronine
FT4	free thyroxine
GCB	germinal center B-cell-like lymphoma
GH	growth hormone
HIV	Human Immunodeficiency Virus
HMB45	Human Melanoma Black 45
IGF-1	insulin-like growth factor 1
IgG4	immunoglobin G4
InDel	Insertion and Deletion
*JAK2*	Janus kinase 2 gene
LH	luteinizing hormone
*LPP*	lipoma-preferred partner gene
MRI	magnetic resonance imaging
Mum-1	Multiple myeloma antigen 1
*MYD88*	Myeloid differentiation primary response 88 gene
NGS	next generation sequencing
NR	normal range
NSE	neuron-specific enolase
NUT	nuclear protein in testis
OS	overall survival
P	progesterone
PAX-5	paired-box domain 5
*PCLO*	Piccolo gene
PCNSL	primary central nervous system lymphoma
*PDCD1LG2*	Programmed cell death 1 ligand 2 gene
PET-CT	positron emission tomography-computed tomography
PFS	progression-free survival
PPL	primary pituitary lymphoma
R²-MTX	Rituximab and methotrexate
R-CHOP	Rituximab-Cyclophosphamide
Hydroxyldaunorubicin	Oncovin and Prednisone
S-100	S-100 protein
SNV	single nucleotide variant
SUV	standard uptake value
Syn	synaptophysin
*TNFRSF14*	tumor necrosis factor receptor superfamily member 14 gene
*TP53*	tumor protein p53 gene
TSH	thyroid stimulating hormone
WES	Whole Exome Sequencing
